# Crosstalk between the actin cytoskeleton and Ran-mediated nuclear transport

**DOI:** 10.1186/1471-2121-6-32

**Published:** 2005-08-24

**Authors:** Svetlana Minakhina, Ron Myers, Marina Druzhinina, Ruth Steward

**Affiliations:** 1 Waksman Institute, Department of Molecular Biology and Biochemistry, NJ Cancer Center, Rutgers University, 190 Frelinghuysen Road, Piscataway, NJ 08854-8020, USA

## Abstract

**Background:**

Transport of macromolecules into and out of the nucleus is a highly regulated process. The RanGTP/RanGDP gradient controls the trafficking of molecules exceeding the diffusion limit of the nuclear pore across the nuclear envelope.

**Results:**

We found genetic interaction between genes establishing the Ran gradient, nuclear transport factor 2 (*ntf-2*), Ran GTPase activating protein (*Sd*), and the gene encoding *Drosophila *Profilin, *chickadee *(*chic*). The severe eye phenotype caused by reduction of NTF2 is suppressed by loss of function mutations in *chic *and gain of function mutations in *Sd *(*RanGAP*). We show that in *chic *mutants, as in *Sd-RanGAP*, nuclear export is impaired.

**Conclusion:**

Our data suggest that Profilin and the organization of the actin cytoskeleton play an important role in nuclear trafficking.

## Background

In eukaryotic cells the nuclear envelope serves as a barrier between the nucleus and cytoplasm. The transport of molecules between the nucleus and cytoplasm occurs through nuclear pore complexes (NPCs). Although some small molecules (<40 kDa) diffuse through the pore, most proteins and RNAs require facilitated transport and special receptors called importins and exportins. This facilitated transport further depends on the small Ras family GTPase Ran (for review see [1,2]).

Similar to other GTPases, Ran is regulated by conformational changes driven by GTP hydrolysis and nucleotide exchange. GDP to GTP exchange happens in the nucleus and is catalyzed by the chromatin-associated protein RCC1 [3]. The GTPase activating protein RanGAP controls RanGTP hydrolysis in the cytoplasm. Spatial partition of these two processes generates a RanGTP concentration gradient across the nuclear pore. This gradient is thought to guide the directionality of nuclear transport.

Protein cargo containing nuclear localization signals (NLS) is recognized by importins and translocates through nuclear pores into the nuclei, where binding to RanGTP causes release of the cargo. In the nuclei, proteins containing nuclear export signals (NESs) form export complexes together with RanGTP. These complexes are then exported, and upon RanGTP hydrolysis in the cytoplasm, the cargo is released. RanGDP nuclear reentry is mediated by the nuclear transport factor 2 (NTF2, [4,5]).

NTF2 was originally identified by its ability to stimulate protein import into nuclei in permeabilized mammalian cells [6]. NTF2 was further shown to have a critical role in actively replenishing the nuclear stock of Ran [4,7,8]. NTF2 catalyzes RanGDP nuclear import, and the concentration of nuclear RanGTP is ultimately increased because of the activity of the RCC1 exchange factor.

Although the RanGTP gradient is required for both import and export, components of the pathway that regulate the gradient have an effect on directionality of cargo nuclear transport. For example, decrease in NTF2 has a primary effect on nuclear import. Conditional alleles of yeast *ntf-2 *show defects in protein nuclear import [9]. Also, depletion of NTF2 using antibodies inhibits nuclear import of NLS-containing proteins in HeLa cells [10]. In Drosophila, partial loss of function of *ntf-2 *affects nuclear import of Rel proteins in immune response and some loss of function alleles show a strong eye phenotype [11,12]. It has been shown in vertebrates that both low levels as well as increased levels of NTF2 impair nuclear import [13-15].

In contrast to NTF2, the intracellular localization of RanGAP appears to be critical for nuclear export [16]. In interphase cells RanGAP is localized to the cytoplasm and a large fraction of the cytoplasmic protein is modified by the ubiquitin related protein SUMO and localized to fibers of the NPCs [17,18]. This strategic position of RanGAP is thought to control the steep concentration gradient of RanGTP across the nuclear envelope.

Mislocalization of RanGAP can reduce nuclear RanGTP levels and lead to reduction of NES-mediated nuclear export, as happens in *Segregation distortion (Sd) *mutants in Drosophila [16]. In *Sd *mutants Sd-RanGAP, an enzymatically active protein refractory to SUMO modification, is expressed in addition to wild-type RanGAP. In these mutants RanGAP is found in higher levels in the cytoplasm and is also detected in nuclei. Sd-RanGAP presumably catalyzes hydrolysis of RanGTP in the nuclei thereby interfering with cargo export. A similar effect can be caused by over-expression of wild-type RanGAP [19].

Ran, in addition to functioning in nuclear-cytoplasmic transport, controls mitotic spindle formation. RanGTP stimulates polymerization of microtubules and RanGAP is found to associate with mitotic spindles [20,21]. Ran also functions in nuclear envelope and nuclear pore assembly [22,23]. These functions require the activities of RanGAP and RCC1, but the contribution of NTF2 is so far unclear.

In a genetic screen we identified mutants in Drosophila Profilin as modifiers of the partial loss of function eye phenotype of *ntf-2*. We find that Profilin is essential for normal nuclear export. This is surprising because the main function of Profilin is to control actin polymerization. RanGAP controls nuclear export and we find that gain of function mutants in *Sd*-*RanGAP *also suppress the *ntf-2 *eye phenotype. Our studies suggest a close connection between the organization of the actin cytoskeleton and nuclear transport.

## Results

### NTF2 and eye development

**Figure 1:**
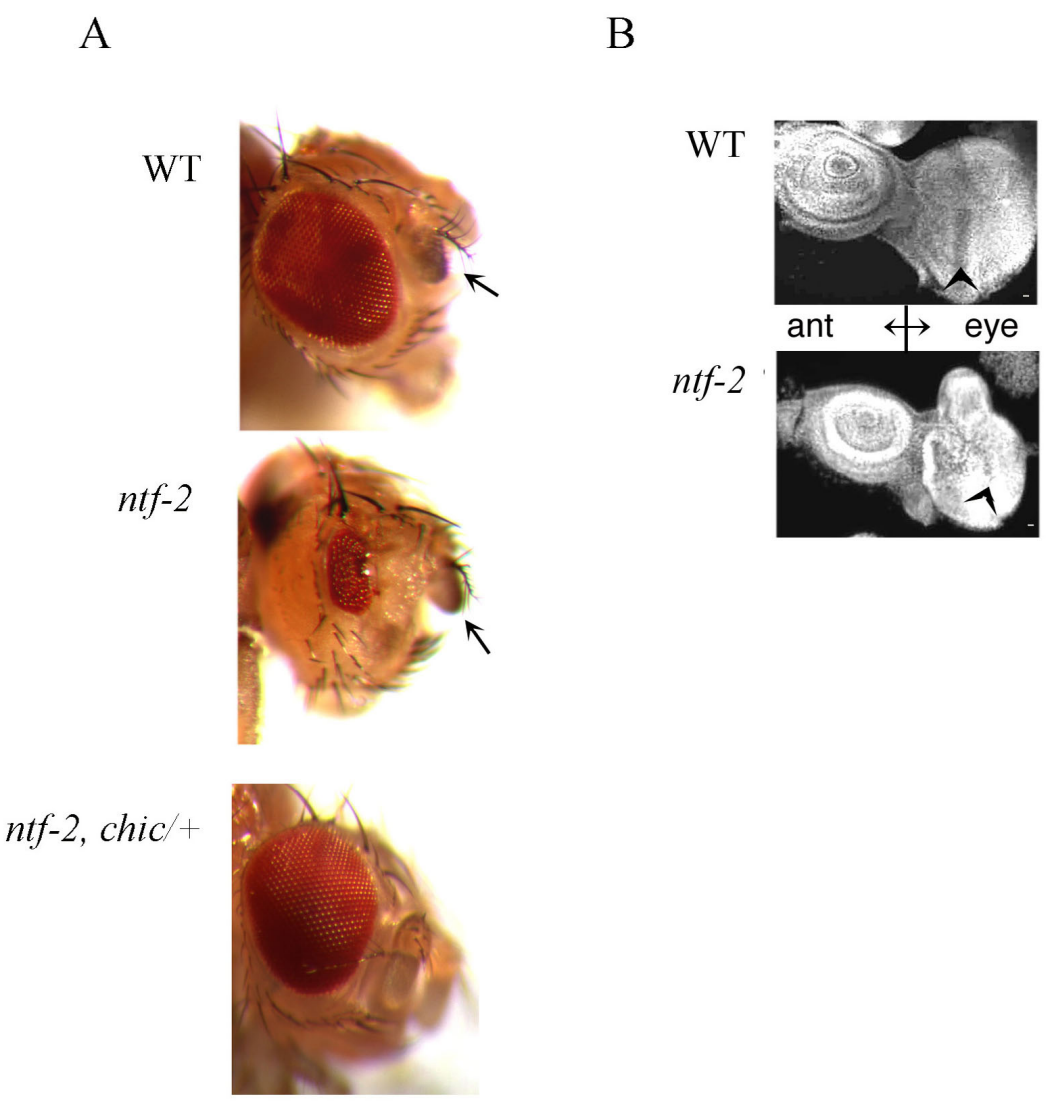
**The *ntf-2 *eye phenotype is rescued by mutants in Profilin (*chic*)**. (A) Wild-type eye, a representative *ntf-2 *eye and the phenotype of a *ntf-2 *eye suppressed by *chic/+*. Note that the antennae (arrow) are normal in mutant animals. (B) Wild-type and *ntf-2 *eye-antennal discs. The antennal discs (ant) are normal in wild-type and mutant, while the *ntf-2 *eye disc (eye) shows abnormal growth and patterning. Size bar represents 10 μm.

*ntf-2 *is an X-linked essential gene. Depending on the allele, animals die between the 2^nd ^larval instar and the pupal stage. Some alleles have an adult survival rate of 8–15% of expected (Table 1), and all survivors show a small eye phenotype, strongly reduced numbers of ommatidia [11,12,24]. The eye phenotype varies from 30% of normal size to a more severe phenotype displaying one or two small patches of 10–40 ommatidia (Fig. 1A).

**Table 1:** Genetic interactions between *ntf-2*, *chic*, and *Sd*. The genetic crosses performed (top of the table). #s in parenthesis indicate columns in the table. The progeny resulting from the cross of *ntf-2/FM7 *females to males carrying the suppressing chromosome were counted. Four alleles of *ntf-2 *are listed in column 1. The genotype of the suppressing chromosomes are presented in the column 2. The numbers of trans-heterozygous females (*ntf-2/+; Su/+*; column 3), of non-mutant males (FM7/Y; Su/+, column 4), and of potentially suppressed mutant males (ntf-2/Y; Su/+) are presented. The *ntf-2/+; Su/+ *males were divided into two columns (5 and 6) depending on their eye phenotype and the viability of these males is indicated (100% X *ntf-2 *males/*ntf-2/+ *females). The number of progeny of the cross shown on the top and marked by * are not shown.

**Parents**							
*ntf-2 *(1)*/FM7c; +/+ *	*×*	*+/Y; Su*(2)*/Balancer*					
**Progeny **							
*ntf-2/+; Su/+*(3)	*ntf-2/Y; Su/+ *(5, 6)						
FM7c/+; Su/+ *	* FM7c/Y; Su/+ *(4)						
ntf-2/+; Balancer/+*	*ntf-2*/*Y; Balancer*/+*						
FM7c/+; Balancer/+*	*FM7c/Y; Balancer/+*						
***ntf-2 *allele (1)**	**Su (2) chromosome Cytology (2)**	***ntf-2/ *+; Su/+ (3)**	**FM7/Y;Su/+ (4)**	***ntf-2/ Y*; Su/+ small eyes (5)**	***ntf*-2/Y; Su/+ normal eyes (6)**	***ntf*-2/Y; viability**	**Suppression of *ntf*-2 eye**

P49	*+ or Balancer*	508	328	72	0	14%	-
P7	*+ or Balancer*	521	325	62	0	12%	-
G0086	*+ or Balancer*	503	154	42	0	8%	-
G0337	*+ or Balancer*	452	180	39	0	9%	-
P49	*Df(2R)Px2 60B; 60D*	50	13	4	19	46%	+
P7	*Df(2R)Px2 60B; 60D*	104	28	10	1	11%	+
P49	*In(2LR)Px[4] 60C-60D,21-22A*	51	14	4	15	39%	+
P7	*In(2LR)Px[4] 60C-60D,21-22A*	155	53	6	1	5%	+
P7	*P{lacW}l(2)04111 [k13009] 22A*	68	24	0	4	6%	+
P49	*Df(2L)cl-h2 25D; 25F*	9	5	0	10	100%	+
P7	*Df(2L)cl-h2 25D; 25F*	23	13	0	13	56%	+
P49	*Df(2L)GpdhA 25D; 26A*	66	14	2	2	6%	+
P7	*Df(2L)GpdhA 25D; 26A*	49	27	1	2	6%	+
P49	*chic*^*K*13321 ^*26A*	53	25	3	1	8%	+
P7	*chic*^*K*13321 ^*26A*	67	40	1	11	18%	+
P49	*chic*^221 ^*26A*	30	12	0	10	33%	+
P7	*chic*^221 ^*26A*	65	48	8	3	17%	+
G0086	*chic*^221 ^*26A*	43	22	0	6	14%	+
G0337	*chic*^221 ^*26A*	69	44	2	4	9%	+
P49	*chic*^01320 ^*26A*	46	35	0	4	9%	+
P49	*chic*^2 ^*26A*	74	68	0	3	4%	+
P7	*chic*^2 ^*26A*	43	33	4	1	12%	+
P49	*In(2R)SD72*, *In(2R)NS*, *Sd[72]*	35	30	0	12	34%	+
G0086	*In(2R)SD72*, *In(2R)NS*, *Sd[72]*	40	7	0	2	5%	+
P49	*UAS-RanGAP12A-6*, *hsp70-GAL4*	16	6	0	5	31%	+
P7	*UAS-RanGAP12A-6*, *hsp70-GAL4*	26	11	0	7	27%	+
P7	*UAS-RanGAP12A-6*, *arm-GAL4*	28	12	1	4	18%	+

**Figure 2:**
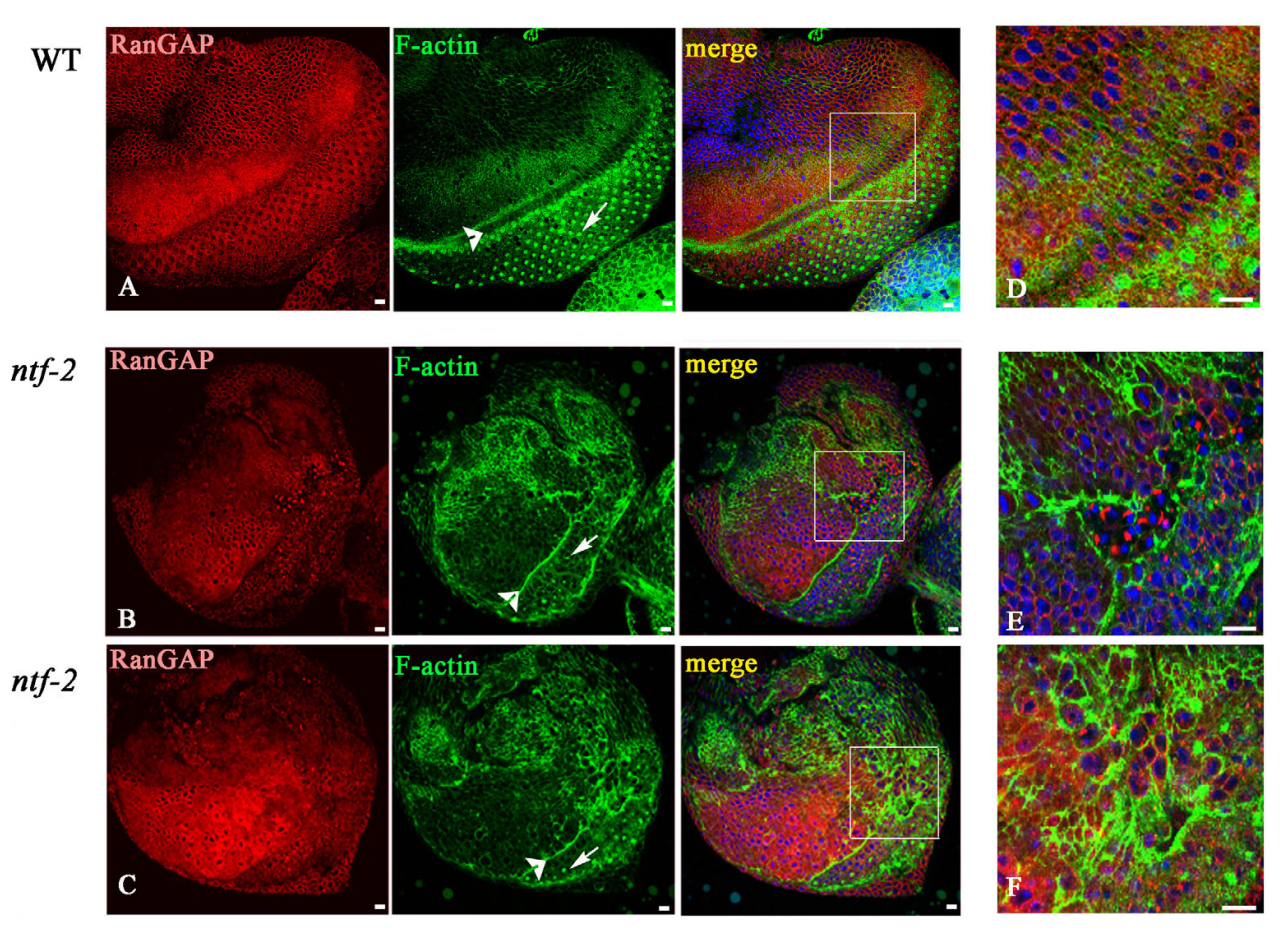
**The *ntf-2 *eye dics are disorganized**. Wild-type eye disc (A, D; arrowhead indicates morphogenetic furrow, arrow indicates rabdomeres). In *ntf-2 *mutants (B, C, E, F) the furrow fails to move and fewer rabdomeres are formed; the organization of the actin cytoskeleton (green) and distribution of RanGAP (red) look abnormal. Squares are magnified in panels D, E, F. In all Figures DNA is shown in blue and the size bar represents 10 μm.

The mutant eye-imaginal discs are smaller than wild-type and are often abnormally shaped (Fig. 1B). Overall, the structure of the mutant eye discs is perturbed and the organization of the actin cytoskeleton is strongly altered (compare Fig. 2A and 2B,C). Only few disorganized, irregularly spaced rabdomere-like structures are apparent in the posterior compartment of the eye disc (arrow in Fig. 2A–C).

### A deficiency screen to identify dominant suppressors of *ntf-2*

We took advantage of the partial loss of function eye phenotype of *ntf-2 *alleles to identify genes functioning with *ntf-2*, and performed a dominant suppressor screen of the eye phenotype. Males from 2^nd ^and 3^rd ^chromosomal deficiency stocks (*deficiency/balancer*) uncovering 70% to 80% of the two autosomes, or about 60% of the *Drosophila *genome, were crossed with *ntf-2*^*P*7^/*FM7 *females (Table 1 top). In the next generation the number of surviving *ntf-2 *males also carrying a deletion was counted and the survivors monitored for their eye phenotype. For our screen we set up 136 individual crosses, many of them repeatedly in order to obtain at least 150 adult progeny to screen for the eye phenotype. We only identified deletions and rearrangements in four regions of the second chromosome that showed suppression (Table 1). The suppression was confirmed using a second *ntf-2 (P49) *allele.

DNA rearrangements affecting regions 22A and 60B-D showed different results with the two *ntf-2 *alleles tested and were not pursued. *Df(2l)cl-h2 *(25D-F) appeared to rescue both viability and the eye phenotype, but the gene responsible for the suppression could not be identified. *Df(2L)GpdhA *(25D-26A) rescued the eye phenotype, but not viability. To identify the gene(s) responsible for the suppression of the eye phenotype we tested mutations in several genes that are uncovered by *Df(2L)GpdhA *and are available from the Drosophila stock center.

Mutants in one gene, *chickadee *(*chic*), encoding Drosophila Profilin [25], uncovered by *Df(2L)GpdhA*, showed suppression of the *ntf-2 *eye phenotype. We tested several loss-of-function alleles of *chic*, including a complete lethal null allele (*chic*^221^) and other partially viable alleles, that are either female, or male and female sterile. All *chic *alleles were crossed with at least 2 *ntf-2 *alleles, except *chic*^221 ^that was tested with 4 different *ntf-2 *alleles. The suppression of the eye phenotype was observed in all crosses and the majority of surviving trans-heterozygous males showed suppression of the *ntf-2 *eye phenotype, restoration of wild-type eyes (Fig. 1A). The percent of males with wild-type eyes varied in different allele combinations. Surprisingly, the eye phenotype was usually either small or wild-type and virtually no eyes of intermediate size were observed.

### Mutations in *chic *(Profilin) affect nuclear export

**Figure 3:**
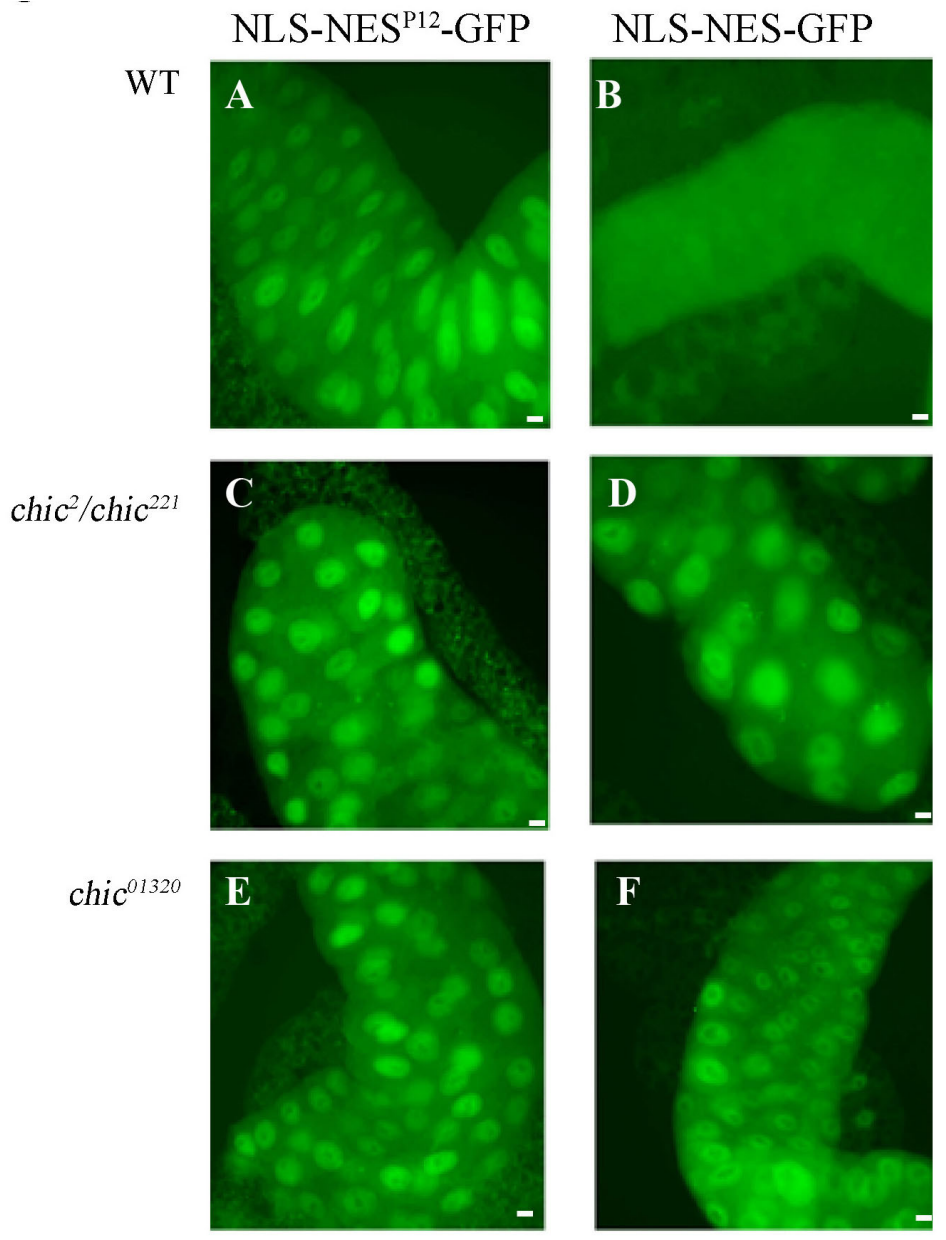
***Chic *impairs nuclear export**. Localization of GFP reporter appended with wild-type NLS and NES (B, D, F) or mutant NES^P12 ^(A, C, E) in salivary gland cells. In wild-type (A) and *chic *mutants (C, E) NLS-NES^P12^-GFP is predominantly localized to the nucleus. In wild-type salivary glands NLS-NES^+^-GFP is mostly found in the cytoplasm (B). In contrast, in *chic *mutants, *chic*^2^/*chic*^221 ^and *chic*^01320 ^homozygotes, NLS-NES^+^-GFP is predominantly nuclear (D, F).

To investigate the cause underlying the suppression of the *ntf-2 *phenotype and possible function of Profilin in nuclear transport, we used a reporter gene approach. We assayed nuclear transport using UAS-NLS-NES reporter constructs C-terminally tagged with GFP in different mutant backgrounds. One construct contains a wild-type NLS and NES (UAS-NLS-NES-GFP), the other a wild-type NLS but a mutant NES that is not recognized by the nuclear export machinery (UAS-NLS-NES^P12^-GFP; [16,26]). Expression of the transgenes was driven by a heatshock-GAL4 driver, and the distribution of GFP was analyzed in salivary glands. As previously shown, the activity of the wild-type NES is stronger then that of the NLS [26]. Hence, in wild-type the NLS-NES-GFP is usually localized in the cytoplasm (Fig. 3B). In contrast, NLS-NES^P12^-GFP has impaired nuclear export and strongly accumulates in nuclei (Fig. 3A). In homozygous *chic*^01320 ^and the hetero-allelic combination *chic*^2^/*chic*^221^, the distribution of the GFP reporter is altered. In contrast to the cytoplasmic distribution of NLS-NES-GFP in wild-type, in the *chic *mutant salivary glands the GFP reporter is found predominantly in the nucleus (Fig. 3D,F). The localization of NLS-NES^P12^-GFP is similar in *chic *and wild-type (Fig. 3C,E), indicating that NLS-mediated import is not affected.

### Sd (RanGAP) suppresses the *ntf-2 *phenotype

RanGAP functions in nuclear export of cargo and in *Sd-RanGAP *mutants the NLS-NES-GFP is found in the nucleus and NLS-NES^P12^-GFP is distributed the same as in wild-type [16,19]. This failure of exporting NLS-NES-GFP in *Sd-RanGAP *mutants is reminiscent of what we observe in *chic *alleles (Fig. 3).

Given the similarity in nuclear export phenotypes in *Sd *and *chic *mutants, we tested if *Sd *would also suppress the eye phenotype of *ntf-2 *alleles. We crossed the Sd (*Sd*^72^, [27]) chromosome with two *ntf-2 *alleles and found that the eye phenotype was suppressed in both of them. To confirm that the *SD-RanGAP *mutation, and not other genes on the Sd chromosome, is responsible for the suppression, we expressed a mutated *Sd-RanGAP *transgene (*UAS-Sd-RanGAP12A-6*; [16]) driven by *hsp70-GAL4 *or *arm-GAL4 *in *ntf-2*^P7 ^and *ntf-2*^P49 ^males and observed similar levels of suppression as seen with *Sd*^72 ^(Table 1).

The genetic interaction between *Sd-RanGAP *and *ntf-2 *is not altogether surprising because both RanGAP and NTF2 are known to function in the formation of the RanGTP-GDP gradient. To investigate if RanGAP is affected in *ntf-2 *mutants we studied the distribution of RanGAP in eye discs.

In wild-type cells Ran-Gap is present in low levels in the cytoplasm and forms a clearly visible punctuated circle around the nucleus (Fig. 2A,D). The punctuate pattern of RanGAP is due to its association with nuclear pores [17,18]. This distribution is different in *ntf-2 *discs. Patches of cells are observed in which RanGAP aggregates in small or large clumps near the nuclei (Fig 2E,F), but in other cells the distribution of the protein looks relatively normal. This observation suggests, that the clumping of RanGAP is an effect of the abnormal organization of the cells within the *ntf-2 *disc. The cells with clumped RanGAP are usually in close proximity to cells with high levels of F-actin.

### Lack of Profilin also affects RanGAP distribution

To investigate a connection between Profilin, RanGAP, and actin, we next asked whether the function of Profilin or actin polymerization might have an effect on RanGAP localization. We generated clones in eye discs of null alleles of the two genes *chic *(*chic*^221^) and, as a control, *act up *(*acu*^*E*636^). Acu participates in actin de-polymerization, the opposite function of Profilin [28].

**Figure 4:**
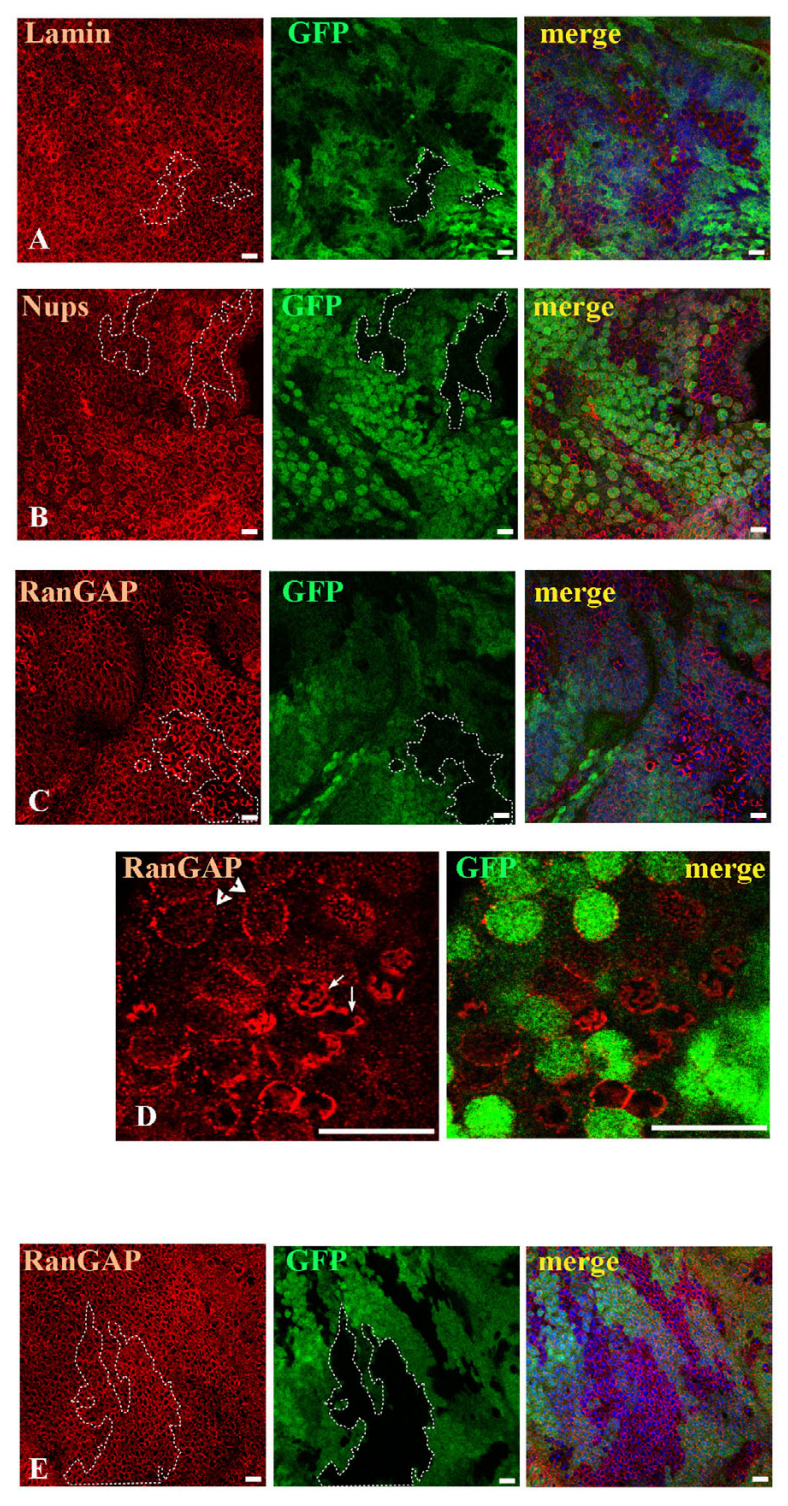
**Localization of RanGAP on *chic *nuclear envelopes is irregular. ***chic*^221 ^eye disc clones show only minor changes in distribution of (A) Lamin (red) and (B) Nups (red). But RanGAP localization (C, D red) is strongly altered appearing more clustered and patchy (arrows) than in wild-type cells, where RanGAP forms uniform dotted rings around nuclei (arrowheads). *act up *loss of function does not affect RanGAP (red) in *acu*^*E*636 ^eye disc clones (E). Mutant clones are marked by the absence of green. The borders of some clones are highlighted with dashed lines.

In *chic *clones RanGAP protein is increased around the nuclear envelope and its distribution is uneven and patchy on the nuclear envelope surface (Fig. 4D arrows). In wild-type even, punctuated circles are observed (arrowheads). This abnormal distribution was found in 100% of examined clones (more then 50). In *chic *clones the level of F-actin was reduced as previously shown [28]. In the *acu *control clones high levels of F-actin are detected as expected (results not shown, [28]), but the distribution of RanGAP is not significantly changed (Fig. 4E).

To test whether this patchy protein distribution of RanGAP on nuclear pores of *chic*^22 ^cells is caused by problems in nuclear envelope assembly, we analyzed the distribution of Lamin and nuclear pore proteins (Nups) in *chic*^221 ^clones (Fig. 4A,B). The distribution of both Lamin and Nups is affected in about 30% of clones. This is likely due to the mislocalization of RanGAP. It has been shown previously that RanGTPase functions in nuclear pore and envelope formation [22,23].

**Figure 5:**
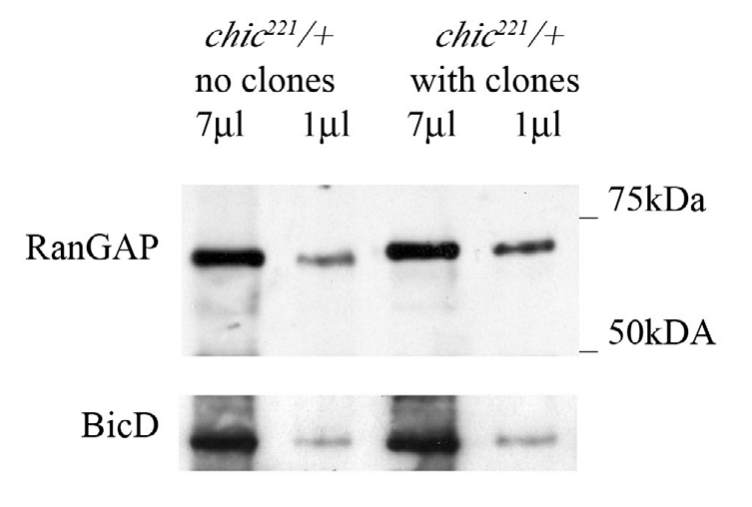
**RanGAP levels in *chic *mutants. **Western blot of extracts from eye-antennal discs and some brain lobes. Compare the amount of RanGAP in *chic*^221^/+ tissue without somatic clones and *chic*^221^/+ with somatic clones; *chic *null tissue, represents 10–30% of eye discs cells and 2–5% of antennae and brain cells. The extracts were loaded at two different concentrations to allow comparison of levels of protein.

The staining experiments show higher levels of RanGAP around nuclei in *chic *eye disc clones. We investigated if this is due to overall higher levels of RanGAP in mutant cells. The *chic *alleles used in the clonal analysis are homozygous lethal therefore we prepared extracts from wild-type and mutant 1^st ^instar larvae. In western blots from extracts of *chic*^221 ^(lethal at first and early second larval instar) and *chic*^01320 ^(viable and female sterile) larvae, the amount of RanGAP present in mutants is not dramatically changed compared to wild-type (results not shown). This may be because RanGAP and Profilin are maternally contributed and therefore at these early stages a difference in levels is not detected. We then dissected eye-antennal discs from normal larvae and larvae with *chic *clones (see experiments shown in Figure 4). The dissected tissues also contained some brain material because eye-antennal discs are next to the brain hemispheres and are difficult to separate. In two separate experiments we see an increase of 30–50% in the intensity of the RanGAP band in extracts from discs carrying *chic*^221 ^somatic clones compared to normal eye discs from *chic*^221^/+ larvae. The intensity of the RanGAP bands were normalized to that of the control Bic-D band and equals 2.6 for discs with clones and 1.8 for wild-type discs (Fig. 5).

## Discussion

NTF2 regulates nuclear import in every cell of the organism. Some *ntf-2 *alleles can produce male sterile but female fertile adults, that all have a striking eye phenotype. This phenotype appears to be caused by lower levels of NTF2 and not an altered protein since alleles showing the eye phenotype have a P-element insertion in the 5' UTR [11,12,24]. The difference in response of tissues to the lower levels of NTF2 is surprising. For instance, the wild-type and mutant antennal discs and antennae appear normal (Fig. 1A arrows), while the eye disc of the mutant has a strongly modified appearance.

We used partial loss of function *ntf-2 *alleles to screen for dominant suppressors of the eye phenotype. Using deletions uncovering more then half of the genome, we identified four regions that can function in the control of the RanGTP-RanGDP balance. While unable to identify specific genes responsible for the dominant interaction in three of these regions, we did find one suppressor, *chic*, encoding Profilin.

We tested several alleles of *chic *with several alleles of *ntf-2 *and observed suppression of the eye phenotype in all combinations. Reducing Profilin suppresses the effects of lower than normal amounts of NTF2. In all cases we observe either small eyes or completely restored eyes similar to wild-type. We detected no intermediate phenotypes, suggesting that a threshold level exists for each protein.

The *ntf-2 *eye phenotype can also be suppressed by a gain of function mutation in *Sd (RanGAP)*. RanGAP regulates the Ran-GTP-to-Ran-GDP balance and is involved in nuclear export. NTF2 controls nuclear import of RanGDP and thus the nuclear trafficking of cargo. Because of the low viability of *ntf-2 *mutants we cannot obtain *ntf-2 *flies expressing a driver and NLS-NES-GFP reporter genes. Hence, the nuclear transport phenotype of *ntf-2 *alleles cannot be determined as we did for *chic *mutants, and was done for *Sd *mutants [16]. Previous investigations in several organisms indicate that in *ntf-2 *mutants, the Ran gradient or its formation is changed affecting cargo import [13-15].

Why lowering the level of Profilin that functions in actin polymerization suppresses the *ntf-2 *phenotype is not immediately apparent, but there are several possible explanations. Lower levels of Profilin may result in reduction of the abnormal actin polymerization in *ntf-2 *mutant eye discs (see Fig. 2). But our finding that the *ntf-2 *eye phenotype is suppressed by the over-expression of RanGAP suggests that the disorganized appearance of F-actin is an indirect result of abnormal nuclear trafficking. Therefore lowering Profilin seems to also affect the abnormal nuclear trafficking inherent to *ntf-2 *eye discs. This supposition is bolstered by our finding that Profilin is essential for normal nuclear export. Our results are consistent with F-actin being regulated by nuclear transport, and in turn, Profilin and Actin controlling aspects of nuclear trafficking.

Unpolymerized actin is found on NPC-attached nucleoplasmic filaments. It has been shown to function in the nuclear export of proteins and RNA [29]. Unpolymerized actin also associates with Profilin and is exported from the nuclei in a Ran-dependant manner [30]. We do not think that these processes have a primary role in our mutant phenotypes because staining of *ntf-2 *eye discs and *chic *clones with anti-actin antibody display no obvious difference in the distribution of non-polymerized actin (results not shown). Nevertheless, these processes have to be considered as part of the crosstalk between the actin cytockeleton and Ran-mediated nuclear trafficking.

That Profilin controls the localization of RanGAP is evident from the abnormal distribution of the protein in *chic *clones. The uneven distribution of RanGAP at the nuclear envelope is not due simply to higher levels of protein. In *Sd *transgenic lines that express wild-type or mutant RanGAP, higher levels of protein are found uniformly distributed in the cytoplasm and nucleus [19]. In *chic *mutant cells, the RanGAP level is about doubled, but the protein distribution is different than that observed in the over-expressing lines.

## Methods

### *Drosophila *stocks and suppressor screen

The *w*^118 ^stock was used as wild-type stock (WT) in all experiments. All fly stocks were obtained from the Bloomington Stock Center [24], except *UAS-RanGAP12A-6*, *UAS-NLS-NES*^*P*12^-*GFP *and *UAS-NLS-NES-GFP *transgenic flies that were sent by Barry Ganetzky and Edwin Chan [16,31].

The following *ntf-2 *stocks were used for suppression experiments: *w ntf-2*^*P*7^/*FM7i*, *P{w [+mC]=ActGFP}JMR3, y[1] f[1], w ntf-2*^*P*49^/*FM7c*, *w ntf-2*^*G*0086^/*FM7c*, *ntf-2*^*G*0337^*/FM7c*. All alleles carry different P-element insertions in the 5' UTR of the gene, 102–112 bases upstream from the start of transcription that cause reduced viability and the eye phenotype (see Table 1, [11,12,24]).

### Suppressor screen

To identify suppressors of *ntf-2*, *ntf-2*^*P*7^/FM7 and *ntf-2*^*P*49^/FM7 virgins were crossed with deficiency males. In the next generation the *ntf-2 *males not carrying a balancer chromosomes were analyzed. We tested 66 deficiencies on the second chromosome and about 70 deficiencies on the third chromosome, and about 25 existing mutations and P-element insertions mapping to the 22–30 cytological region of chromosome two. All were obtained from the Bloomington Stock center.

Alleles of chickadee, *chic*^*K*1332^, *chic*^221^, *chic*^01320^, *chic*^2^, *In(2R)SD72*, *In(2R)NS*, *Sd[72]*, GAL4 drivers and Balancer chromosomes were obtained from Bloomington Stock Center. *UAS-RanGAP12A-6*, UAS-NLS-NES^*P*12^-GFP and UAS-NLS-NES-GFP transgenic flies were kindly sent to us by Barry Ganetzky and Edwin Chan [16].

### Nuclear transport assay

To identify mutant animals at larval stages we used the GFP marked balancers *FM7i P{w [+mC]=ActGFP}JMR3 *and *CyO*, *P{w [+mW.hs]=Ubi-GFP.S65T}PAD1*. Mutant 2^nd ^instar larvae were distinguished from their heterozygous siblings by the absence of green fluorescent protein. The expression of GFP-reporter genes were induced by 1 h incubation at 36°C. To diminish the possible effect of heating on nuclear trafficking we waited for 24 h before dissecting and fixing salivary glands. Localization of GFP was observed and fluorescence images obtained using a Zeizz Axioplan 2 (Zeiss) microscope and Image Pro Plus software.

### Eye disc clones

*FRT40 chic*^221 ^and *FRT40 acu*^*E*636 ^chromosomes were obtained from Jessica Treisman. Clones in eye discs were generated as described [28].

### Antibodies

Rabbit polyclonal antibody against Drosophila RanGAP was provided by Sinthia Stabber and Barry Ganetzki [16], mab414 recognizing nucleoporins with FXF repeats [32,33] was obtained from BAbCO (Richmond, CA), and anti-Lamin antibody was a gift from Paul Fischer, Stonybrook. Western analysis was performed as previously described [16,17].

### Immunostaining

For antibody staining 3rd instar larvae were inverted in phosphate-buffered saline (PBS) and immediately fixed in 4% paraformaldehyde in PBS with 2% DMSO for 40 min and washed several times in PBT (PBS, 0.1% Triton X-100). Then tissues were blocked for 2 hours in PBS containing 1% bovine serum albumin (BSA) and 1% Triton X-100. Antibody incubations were done in PBT with 1% BSA overnight at 4°C. Anti-RanGAP rabbit serum was used at a 1:1000 or 1:800 dilution, and anti-RanGAP-1 monoclonal antibody was used at a 1:400 dilution, mab414 was used at a 1:300 dilution, anti-Lamin – 1:30. Secondary antibodies were used at a dilution of 1:500. Cy-3 conjugated anti-mouse and anti-rabbit IgG were purchased from Jackson Immuno Research Laboratories Inc. (West Grove, PA). F-actin was visualized by incubation with Alexa488 Phalloidin at 1:80 for 2 hours. DNA was stained using Hoechst 33258 (Molecular Probes, Eugene, OR). Samples were mounted in Vectashield (Vector Laboratories) and examined with a Leica DM IRBE (Leica) laser scanning confocal microscope. The images were analyzed with Leica Microsystems software and further processed using Adobe PhotoShop.

## Authors' contributions

SM participated in the design of the study, and was involved in all experiments presented. RM carried out the suppressor screen and characterized *chic *as a suppressor of *ntf-2*. MD carried out the immunoassays. RS conceived the study, worked on the genetic screens. SM and RS wrote the paper. All authors read and approved the final manuscript.
